# Symmetry, Entropy, Diversity and (Why Not?) Quantum Statistics in Society

**DOI:** 10.3390/e21020144

**Published:** 2019-02-03

**Authors:** Jorge Rosenblatt

**Affiliations:** Institut National de Sciences Appliquées, 20 Avenue des Buttes de Coësmes CS 70839, CEDEX 7, F35708 Rennes, France; jrosenblatt@wanadoo.fr; Tel.: +33-(0)-1-4655-5574

**Keywords:** entropy production, quantum-like systems, econophysics, indistinguishability, inequality, non-extensive entropy

## Abstract

We describe society as an out-of-equilibrium probabilistic system: in it, N individuals occupy W resource states and produce entropy S over definite time periods. The resulting thermodynamics are however unusual, because a second entropy, H, measures *inequality* or *diversity*―a typically social feature―in the distribution of available resources. A symmetry phase transition takes place at Gini values 1/3, where realistic distributions become asymmetric. Four constraints act on S: N and W, and new ones, *diversity* and interactions between individuals; the latter are determined by the coordinates of a single point in the data, the peak. The occupation number of a job is either zero or one, suggesting Fermi–Dirac statistics for employment. Contrariwise, an indefinite number of individuals can occupy a state defined as a quantile of income or of age, so Bose–Einstein statistics may be required. *Indistinguishability* rather than anonymity of individuals and resources is thus needed. Interactions between individuals define classes of equivalence that happen to coincide with acceptable definitions of social classes or periods in human life. The entropy S is non-extensive and obtainable from data. Theoretical laws are compared to empirical data in four different cases of economic or physiological diversity. Acceptable fits are found for all of them.

## 1. Introduction

In previous papers [1,2], we fitted Lorenz inequality curves [3]―non-thermodynamic quantities at first sight―with a simple model of social entropy. Symmetric distribution laws predict equal probabilities of being in the oldest or youngest decile, or to belong either to the richest or the poorest one. In fact, important differences between such deciles are found practically everywhere, and this requires, as shown below, Gini [4] coefficients Gi≥1/3. The assumption of a symmetry phase transition, similar to that in binary alloys [5,6] and superconductors [7], provides very good fits to data [1]. Four cases in this paper hint indeed at asymmetric distributions as the real-world rule.

Entropy can measure social diversity or inequality [8,9], and possibly other “qualitative” quantities like difficulty, ability [10] or sensitivity. Societies cannot exist without a minimum of cohesion among their members, that is, individuals are correlated through an attractive relation. Interactions are successfully taken into account in the present paper. In principle, they may affect the definition of inequality indicators. The latter are expected to satisfy certain conditions such as anonymity [11], that is, all permutations of individuals or their resources are equivalent and count for one. We discuss the statistical consequences of this conjecture.

Whether societies are or are not in equilibrium is a relevant question in any theoretical approach [12]. They have been visualized as nonequilibrium dynamic networks of voters [13]. A significant difference between them results here from the assumption of a symmetry phase transition in a previous paper [1]. A comparison of predictions against real data is required, and is the object of this paper.

Societies evolve, produce and consume, and therefore we describe them here as nonequilibrium, interacting, entropy producing and asymmetrically distributed statistical systems with a large number of degrees of freedom. We apply the resulting theory to the prediction of data in the previous paper: they display a rather large variety of fitting parameters and ranges of interaction, necessary to test model forecasts [1]. Two types of data are fitted, economic and demographic, and two examples of diversity are discussed in each of them: incomes in the USA [14] and *per capita* electricity consumption [15] in 170 countries illustrate the first case; life expectancy up to one hundred years [14] and survival after cancer [16], describe the second one. Data allow a calculation of the average entropy production during periods of five years for cancer, one year in the other cases. Correlations are due to interactions between individuals or similarities in age periods of human life. Dollars, kWh, years of life expectancy or of age without cancer, thus become resource or benefit units (BUs) here. Individuals may refer to persons, households, economic agents, countries, and so on. We bridge the gap between individual situations and global social pictures, using the following concepts and, particularly, their interplay.

(*i*) *States.* The state of an individual is defined as the amount of resources (of a single type in this paper) available to him or her during a specified period. Our data involve the number of individuals populating a quantile of such states. We could equally well refer to the state of a benefit, describing the fraction of total resource allotted to a quantile of the population. Dirac’s notation is useful to define individual or benefit configurations: 〈X|k〉 means “individual 〈X| occupies state |k〉”, while 〈x|j〉 means “resource 〈x| is allotted to state |j〉”. The number of states does not necessarily coincide with that of individuals, N (think of jobs as states and workers as individuals), and the same can be said of the total number of benefit states and total resource W. We form groups of states following arbitrary criteria (for example, deciles), or such groups may be spontaneous, that is, socially generated, like the middle class or the adult population. Let $N_{k}$ be the number of individuals in group k (k=1, 2, …K) and $G_{k}$ the number of states in it. The average group occupation numbers are $\nu_{k}=N_{k}/G_{k}$, components of a vector ν. Resources $w_{k}$, divided by the average $\bar{w}=W/N$, provide the components of a vector $\omega =w/\bar{w}.$ A connection between ν and ω finally leads to the distribution law ν(ω).

(*ii*) *Entropy* is a sum over states. In isolated systems, with no other constraint than the obvious one that probabilities add up to unity, it reaches its maximum (equilibrium) value when individuals occupy all accessible states with equal probability, that is, when the distribution is uniform, therefore *symmetric*. A very particular case is equal resources, that is, a δ-function distribution law, usually taken as a reference state for Lorenz curves. Social processes are irreversible, societies actually *produce* entropy S(ν) over a given period of time.

(*iii*) *Inequality* or *diversity* is a specifically social parameter. It is measured here by the extropy [17], i.e., the entropy *produced* [18] by an initially out-of-equilibrium system as it evolves towards equilibrium, that is, max(H)−H(ω). In this way H(ω) provides both a measurement of inequality and a constraint on social entropy production S(ν). The normalised version of the extropy, 1−H(ω)/max(H), is just the redundancy of information theory [19].

(*iv*) *Symmetry.* Children tend to have a longer life expectancy than their parents, and the poor are more numerous than the rich. Populations therefore have *asymmetrical* nonequilibrium distributions, and this is the case of all real-data situations discussed here. Symmetry and symmetry transitions are thus relevant, at least in social and demographic contexts.

(*v*) *Correlations*. Specific periods in human life, as well as interactions between individuals, are assumed here to establish correlations between them. Interactions are here supposed to be reflexive, symmetrical and transitive, which is just the definition of classes of equivalence. In fact, they will be seen to coincide with acceptable descriptions of social classes or periods like childhood or oldness. Moreover, members of a class cluster naturally, which implies attractive intraclass interactions. Interclass correlations and non-additive entropies [20,21] finally furnish a convenient picture of social systems.

(*vi*) *Indistinguishability.* Statistical descriptions of employment and incomes may be drastically different. A Fermi–Dirac (F–D) statistic applies to employment states, just because the number of individuals on a job is either zero or one. Alternatively, if states are specified as quantiles of income, the upper limit to the amount of benefit in any of them is total resource, which pleads for Bose–Einstein (B–E) statistics. Social and economic laws are thus expected to be invariant against exchange [22]―rather than permutation―of two indistinguishable―rather than anonymous―individuals or resources.

Mathematical functions are assumed to fulfil the conditions of continuity, differentiability, and so on, required to perform indicated operations on them. We mark conceptually important conjectures by the letter “C” followed by an ordinal. *Indistinguishability* means then (C1) that social phenomena admit a quantum-like statistical description. Incidentally, other cases exist where classical ^entities^ [23,24] obey quantum statistics.

Section 2 discusses the relation between social states and entropy. Section 3 dwells on fictitious societies of independent individuals, and Section 4 examines an inequality- and interaction-dependent model providing rather good fits to actual data. Conclusions appear in Section 5.

## 2. Symmetry, Entropy and Universality

Let F(ω) be the cumulative population fraction (CPF) and L(F) the cumulative benefit fraction (CBF). The Gini coefficient is, by definition,

(1) $$Gi=2\int_{0}^{1}\left(F-L\left(F\right)\right)dF=1-2\int_{0}^{1}L\left(F\right)dF=1-2〈L\rangle .$$

That is, all Lorenz curves having the same value of 〈L〉 have the same Gini coefficient. In particular, symmetric distributions should be Gini-equivalent to uniform distributions, with maximum and minimum benefits $\omega_{M}$ and $\omega_{m}$, respectively. Define $R_{u}=\omega_{m}/\omega_{M}\geq 0$: the uniform probability density function is $f_{u}\left(\omega \right)=1/\left(\omega_{M}-\omega_{m}\right)$ when $\omega_{M}\geq \omega \geq \omega_{m}$, zero otherwise, with CPF $F_{u}\left(\omega \right)=\left(\omega -\omega_{m}\right)/\left(\omega_{M}-\omega_{m}\right)$. The corresponding CBF gives

(2) $$Gi=1-2\int_{0}^{1}\frac{2R_{u}F_{u}+\left(1-R_{u}\right)F_{u}{}^{2}}{1+R_{u}}dF_{u}=\frac{1}{3}\frac{1-R_{u}}{1+R_{u}}\leq \frac{1}{3}.$$

Perfect equality results when $R_{u}=1$ and $L_{u}=F_{u}$. Symmetric distributions lead instead to a maximum of inequality when $R_{u}=0$ and thus $L_{u}=F_{u}{}^{2}$. Symmetry is just incompatible with Gi>1/3. As shown in [1], symmetric distributions have $\omega_{m}+\omega_{M}=2$, while asymmetry imposes $\omega_{M}>2$, actually the usual case in real life. Now, since asymmetric distributions and Gini values above 1/3 do exist, a symmetry change―a phase transition―must take place, which is expected to be at Gi=1/3. Experimental evidence supports these results: Figure 3 in reference [25], dealing with size distributions of beer bubbles, shows a great number of Gini coefficients above 0.33, and none below, showing that symmetric distributions are indeed unlikely. In a phase transition, *universality* is expected, whereby near the transition thermodynamic quantities and their possible social counterparts are generalised homogeneous functions [26] of their arguments. We apply this condition to social welfare U(w;W,N) [11].

### 2.1. Welfare, Inequality and Symmetry

Social welfare must increase with W and decrease as 1/N when the population increases but total benefit remains constant. Generalised homogeneity means then that transformations W→aW and N→bN reduce to multiplication of both U(·) and w by a/b. That is, a=1/W and b=1/N require

(3) $$U\left(w;W,N\right)=\frac{b}{a}U\left(\frac{a}{b}w;aW,bN\right)=\frac{W}{N}U\left(\frac{w}{\bar{w}};1,1\right)=\bar{w}\;U_{0}\left(\omega \right).$$

Equation (3) compels the independent variable in $U_{0}\left(\cdot \right)$ to be $\omega =w/\bar{w}$. Social welfare should decrease as inequality increases, a condition satisfied by Foster and Sen’s [27] proposal, where $U_{0}\left(\omega \right)=1-I\left(\omega \right)=H\left(\omega \right)/\max\left(H\left(\omega \right)\right)$ and I(ω) is the normalised measure of inequality. Properties of inequality indicators [11] easily follow from the fact that ω, and therefore I(ω), are scale-, replication- and permutation-invariant, that is, they do not change if all benefits are multiplied by the same positive constant, the distribution is replaced by a number of replicas of itself, or the ordering of components of the vector ω is changed. Coincidence of economical and statistical approaches reinforces the present one.

### 2.2. Interactions

Phase transitions reveal relevant interactions in both thermodynamic and social systems. Assume then that individuals occupy sites $r_{i}$ in a periodic lattice embedded in a Euclidean space of dimensionality d. Interaction links between individuals are randomly established. We measure distances $r_{ij}=\left|r_{i}-r_{j}\right|$ in this space in units of nearest-neighbour distance and assume correlations to exist and to decrease with distance as $\mathrm{corr}\left(r_{i},r_{j}\right)\sim r_{ij}{}^{-\delta }$, with δ positive. Such is the case in percolative clusters. In a qualitative approach [21], consider constant-density groups: an individual out of N in a cluster of linear size $R\sim N^{1/d}$ interacts with $\int_{1}^{R}r^{d-1-\delta }dr=\left(1-N^{-\theta }\right)/\theta d=\ln_{\theta }N/d$ other individuals, where θ=(δ/d)−1 and $\ln_{\theta }N\underset{\theta \to 0}{\to }\ln N$. We refer to functions $\ln_{\theta }\left(\cdot \right)$ as quasi-logarithms. If θ is positive, when N goes to infinity the number of interactions per individual remains finite, of the order of 1/θd=1/(δ−d)≥0. This defines short-range correlations: society behaves as an assembly of independent finite clusters, with possible inner interactions. Long-range correlations, where each individual is connected to infinitely many others as N grows without limit, occur for θ≤0. The parameter θ thus conveys information on the existence, the range and, as we shall show in Section 4.1, the strength of many-body interactions. We point out that $\ln_{q}\left(\cdot \right)$, where q=θ+1=δ/d, is a more usual notation for quasi-logarithms.

### 2.3. Classical Independent Individuals

Contrary to the preceding Section, assume that society is composed of noninteracting anonymous individuals, whose permutations count for one. They form K groups of $N_{k}$ individuals each; the entropy is that of Maxwell, Boltzmann and Gibbs (MBG), $S_{MBG}=\ln\Gamma_{MBG}=\ln\left(\sum_{\left\{N\right\}}\frac{N!}{N_{1}!N_{2}!\ldots N_{K}!}\right)$. The symbol {N} means that *each* term in the sum satisfies $\sum_{k}N_{k}=N$. This is similar to the case of gases in physics, although the latter are not affected by inequality. It in fact becomes relevant in social systems. Its measure is given by the MBG entropy $H_{MBG}=\ln\Omega_{MBG}=\ln\left(\sum_{\left\{W\right\}}\frac{W!}{W_{1}!W_{2}!\ldots W_{K}!}\right)$ in $U_{0}\left(\omega \right)$, Equation (3). A simple textbook exercise [21] shows that this is not the right way to count configurations in social systems: quantum statistics are necessary.

#### 2.3.1. Paradoxical Distinguishability

Count the states in an elementary society consisting of two distinguishable individuals, A and B, two equally distinguishable BUs labelled a and b, and G=C=3 states, numbered k=1, 2, 3. Employment states result from three jobs that individuals can occupy or not, and where available resources can alight. If states are defined by income, the amount of resource in each of them is arbitrary. We use Dirac’s notation as discussed in the Introduction. An equal sign relates equivalent configurations (all permutations count for one), while the sign “⇔” indicates their *indistinguishability* (the statistic of independent individuals is either F–D or B–E). The MBG expressions imply that N=W=2 such individuals or BUs populate three states in $\Gamma_{MBG}=\Omega_{MBG}=\sum_{\left\{2\right\}}\frac{2!}{W_{1}!W_{2}!W_{3}!}=\sum_{\left\{2\right\}}\frac{2!}{N_{1}!N_{2}!N_{3}!}=9$ ways. A paradoxical result in more than one sense, as we now show.

##### Employment Paradox

Assume anonymity, that is, all permutations of individuals A and B are equivalent and count for one [11]. Which is one too many for employment states, because $\Gamma_{MBG}$ involves configurations of the type 〈A|k〉+〈B|k′〉=〈B|k〉+〈A|k′〉, with k=k′, that is, where individuals A and B occupy the same job. The notion of state shows here its relevance: anonymity ignores the fundamental zero-or-one restriction on the occupation of employment states. Only three states instead of nine are possible if 〈A|k〉+〈B|k′〉⇔〈A|k′〉+〈B|k〉 satisfies the condition k≠k′. This is similar to Gibbs paradox in classical statistical physics. A F–D statistic furnishes the right value for employment: $G_{k}$ possible states result in $\Gamma_{FD_{k}}=G_{k}!/N_{k}!\left(G_{k}-N_{k}\right)!$ instead of $\Gamma_{MBG}$.

##### Resource Paradox

Five ten-unit banknotes are physically distinguishable from a single bill of fifty units, but they are socially indistinguishable. Individual states have total benefit as an upper limit of income, so this type of resource obeys B–E statistics. Three states |k〉 involve therefore six possible configurations instead of nine, of the type 〈a|k〉+〈b|k′〉⇔〈a|k′〉+〈b|k〉⇔〈2a|k k′〉, where k=k′ is now included. Combinatorics gives the number of configurations for $C_{k}$ benefit states as

(4) $$\Omega_{BE_{k}}=\frac{\left(W_{k}+C_{k}-1\right)!}{W_{k}!\left(C_{k}-1\right)!}\;\approx \frac{\left(W_{k}+C_{k}\right)!}{W_{k}!C_{k}!}\;.$$

The second Equation (4) applies when $C_{k}\gg 1$.

##### Individuals’ Paradox

Social individuals, like resources, are B–E indistinguishable, and an equation similar to (4) should apply. Indeed, one finds $\Gamma_{BE}=6$ for our elementary society. With $G_{k}$ states and $N_{k}$ individuals in group k, we have, in general:

(5) $$\Gamma_{BE_{k}}=\frac{\left(N_{k}+G_{k}-1\right)!}{N_{k}!\left(G_{k}-1\right)!}\approx \frac{\left(N_{k}+G_{k}\right)!}{N_{k}!G_{k}!}.$$

The second equation applies when $G_{k}\gg 1$. Statistics for elementary particles result from their spin and are therefore an intrinsic particle property, but they depend on the nature of individual states in social systems. The same individuals may obey F–D employment statistics and display B–E behaviour when their incomes are at stake.

### 2.4. Unattainable Dilution

Is there a connection between the number of states $C_{k}$ (Equation (4)) and the number of individuals $N_{k}$ (Equation (5)) in spontaneous groups? Classical statistics requires a high degree of dilution, that is, many more benefit states than individuals, $N_{k}/C_{k}\ll 1$. This would mean, for examples discussed here, many more jobs than employees, or life expectancy at birth well above one hundred years. In actual fact, these quantities are not strictly equal but of the same order of magnitude, $C_{k}\approx N_{k}=G_{k}\nu_{k}$. This asserts the impossibility of dilution. We therefore conjecture that the attractive intraclass interaction, referred to in the Introduction—a many-body effect—is strong enough to induce high occupancy of available benefit states. This amounts to a new formulation of C1, C1’, in the particular case of spontaneous groups: Dilution is impossible for such groups, they never behave classically.

## 3. Entropic Duality

Apply C1’ and Stirling’s large number formula to Equation (4). One obtains a dimensionless measure of social equality given by the out-of-equilibrium B–E entropy [28] with $G_{k}\nu_{k}$ states in group k:

(6) $$H_{BE}\left(\omega \right)=\overset{K}{\underset{k=1}{\sum }}\ln\Omega_{BE_{k}}=\overset{K}{\underset{k=1}{\sum }}C_{k}h_{BE}\left(\omega_{k}\right)\approx \overset{K}{\underset{k=1}{\sum }}G_{k}\nu_{k}h_{BE}\left(\omega_{k}\right),$$

to which the group contribution is

(7) $$h_{BE}\left(\omega_{k}\right)\;=\left(1+\omega_{k}\right)\ln\left(1+\omega_{k}\right)-\omega_{k}\ln\omega_{k}$$

Social individuals produce in turn ν-dependent entropy resulting from Equation (5):

(8) $$S_{BE}\left(\nu \right)=\overset{K}{\underset{k=1}{\sum }}\ln\Gamma_{BE_{k}}=\overset{K}{\underset{k=1}{\sum }}G_{k}s_{BE}\left(\nu_{k}\right),$$

where the B–E entropy production by $\nu_{k}$ individuals on $G_{k}$ states is

(9) $$s_{BE}\left(\nu_{k}\right)=\left(1+\nu_{k}\right)\ln\left(1+\nu_{k}\right)-\nu_{k}\ln\nu_{k}$$

Equation (9) applies equally well to equilibrium and nonequilibrium entropies, but of course the values of $\nu_{k}$ in each case are different. The functional relation ν(ω) requires still another conjecture, C2: The most probable path for entropy production maximises the number of ways of reaching the final distribution, and thereby the socially constrained entropy production $S_{\mathrm{BE}}\left(\nu \right)$ during the period of interest [29].

### 3.1. Constraints

Two constraints are obvious, $N=\sum_{k=1}^{K}G_{k}\nu_{k}$ and $W/\bar{w}=\sum_{k=1}^{K}G_{k}\nu_{k}\omega_{k}$. Lagrange multipliers should thereby result in two adjustable parameters, namely α and β. But the out-of-equilibrium value of the entropic form $H_{BE}\left(\omega \right)$, due to society’s self-inflicted inequality, is a third constraint. An additional Lagrange multiplier is necessary, and results in a new parameter, λ, measuring diversity. According to C2 and Equations (6) to (9), entropy production $s_{BE}\left(\nu_{k}\right)$ obeys

(10) $$\frac{\partial }{\partial \nu_{k}}\left[s_{BE}\left(\nu_{k}\right)-\nu_{k}\left(\beta \varphi_{BE}\left(\omega_{k},\lambda \right)+\alpha \right)\right]=\ln\left(1+\frac{1}{\nu_{k}}\right)-\left[\beta \varphi_{BE}\left(\omega_{k},\lambda \right)+\alpha \right]=0,$$

that is, the first term in the second Equation (10) is a linear function of the social free energy per individual:

(11) $$\varphi_{BE}\left(\omega ,\lambda \right)=\omega +\lambda \left[\left(1+\omega \right)\ln\left(1+\omega \right)-\omega \ln\omega \right]$$

formally similar to the Helmholtz free energy per molecule of a B–E ideal gas at “temperature” −λ; as shown in the next Section, this quantity is positive. The distribution law for noninteracting individuals is:

(12) $$\nu_{BE}\left(\omega ;\alpha ,\beta ,\lambda \right)=\frac{1}{\exp\left(\beta \varphi_{BE}\left(\omega ,\lambda \right)+\alpha \right)-1}.$$

In case of a F–D statistic for individuals, but no change in the B–E nature of resources, it becomes

(13) $$\nu_{FD}\left(\omega ;\alpha ,\beta ,\lambda \right)=\frac{1}{\exp\left(\beta \varphi_{BE}\left(\omega ,\lambda \right)+\alpha \right)+1}.$$

A similar procedure would apply to any number of independent resources, as many entropic forms $H_{BE,i}$, parameters $\lambda_{i}$ and several peaks in the distribution. Our data show however a single peak in $\nu_{BE}\left(\omega \right)$ at $\omega =\omega_{p}$: it is a poverty peak for income, a youth peak for life expectancy and an old-age peak for cancer incidence. It coincides with a minimum of the social free energy.

### 3.2. Parameters

Since Equation (11) implies that $\varphi_{BE}\left(0,\lambda \right)=0$ for any finite λ, α>0 determines the fraction of population $1/\left(e^{\alpha }-1\right)$ suffering from extreme poverty or very short life expectancy, that is, ω≈0; α=−βμ, where μ<0 is the counterpart of the chemical potential in physics. The peak abscissa $\omega_{p}$ defines λ through

(14) $$\omega_{p}\left(\lambda \right)=\frac{1}{e^{-1/\lambda }-1},\;\lambda \left(\omega_{p}\right)=-\frac{1}{{\frac{dh_{BE}}{d\omega }|}_{\omega =\omega_{p}}}=-\frac{1}{\ln\left(1+\frac{1}{\omega_{p}}\right)}.$$

Now, ω must be positive because nobody can survive without resources. This is in particular the case of $\omega_{p}$, so Equation (14) shows that only negative values of $\lambda \left(\omega_{p}\right)$ are realistic. Parameters β and α result from linear regression once λ has been determined; $1/\beta =〈\omega \rangle +\lambda 〈h_{BE}\left(\omega \right)\rangle =〈\varphi_{BE}\left(\omega ,\lambda \right)\rangle$ plays the role of absolute temperature.

### 3.3. Anonymity

In the classical limit, the additional constraint becomes $H_{MBG}\left(\omega_{k}\right)=\sum_{k}^{K}G_{k}\nu_{k}\omega_{k}\left(1-\ln\omega_{k}\right)$. In place of Equation (12) one obtains $\nu_{MBG}\left(\omega ,\lambda_{MBG}\right)=\exp\left\{-\left[\beta_{MBG}\varphi_{MBG}\left(\omega ,\lambda_{MBG}\right)+\alpha_{MBG}\right]\right\}$. Entropy production is given in such a case by $S_{MBG}\left(\nu_{k}\right)=\sum_{k}^{K}G_{k}\nu_{k}\left(1-\ln\nu_{k}\right)$. We get, in place of Equation (9), $\varphi_{MBG}\left(\omega ,\lambda_{MBG}\right)=\omega \left(1+\lambda_{MBG}\ln\omega \right)$. The poverty peak is found at $\omega_{MBG,p}=\exp(1/\lambda_{MBG})$.

## 4. Results

**Figure 1:**
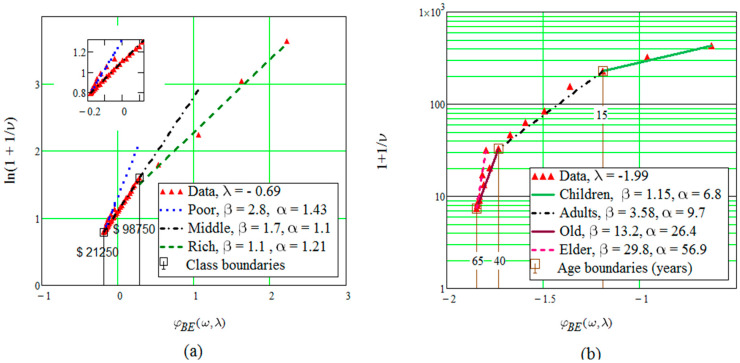
Two fits of smoothed data on: (**a**) Incomes in the USA, (**b**) Benefit decreases when the number of cancer cases increases.

The distribution of incomes in the USA shows apparently spurious oscillations, with local maxima and minima that happen to coincide with tax return brackets. Numerical smoothing was necessary, and it was applied to all distributions to warrant equality of treatment. For the same reason fitting was also sought for smoothed curves, which had anyway little effect on resources other than incomes. Equation (10) assumes independent individuals and refers to the whole distribution. It therefore predicts a single straight line for plots like those in Figure 1, where Figure 1a refers to annual incomes per household in the USA, the insert being an enlargement of the low-income region; and Figure 1b illustrates cancer incidence on male population in New York City. Now, Figure 1a displays three segments instead of one, and Figure 1b shows four segments. Relevant ages and middle-class income boundaries are indicated and look reasonable. Not shown, life expectancy also displays four segments, with age boundaries close to those in Figure 1b, but in reverse order; 13, 45 and 65 years. Electricity consumption requires five segments, to be discussed in Section 4.2.

Segments in Figure 1 provide a piecewise fit of Equation (10). This is compatible with the picture of finite clusters as described for short-range interactions in Section 2.2. The point here is that each segment clearly corresponds to a social class in Figure 1a and well-known periods in human life in Figure 1b. Populations economically or physiologically wealthier, when ordered by increasing benefit, show decreasing slopes in Figure 1. This results from the definition of absolute “temperature” that follows Equation (14), for segments fitting “hotter” (richer or younger) fractions of society. Their slopes thus describe intraclass behaviour. Possible interclass interactions are discussed in the next Section. Greater equality implies a decrease in the number of states per individual in a group, $1/\nu_{k}=G_{k}/N_{k}$.

The fraction of population $F_{p}=\mathrm{Pr}\left(\omega \leq \omega_{p}\right)$ objectively defines the poorest or the oldest in the distribution. Dissimilar data like ages and social classes provide similar results and so plead for common treatment of different types of diversity.

### 4.1. Interacting Classes

Can the theory feature slope changes and thereby interactions? Consider, for instance, a three-class system like that in Figure 1a, and independent quantities x,y,z, with probabilities $p_{x},\;p_{y},\;p_{z}$, respectively, so total probability is $p_{xyz}=p_{x}p_{y}p_{z}$, with θ=0. The replacement of logarithms by quasi-logarithms results in products that should be responsive [20,21] to interclass correlations:

(15) $$\begin{array}{ll} \ln_{\theta }\left(p_{xyz}\right)= & \ln_{\theta }p_{x}+\ln_{\theta }p_{y}+\ln_{\theta }p_{z}\\ & \;\;\;\;\;+\left(-\theta \right)\left[\ln_{\theta }p_{x}{\;\ln}_{\theta }p_{y}+\ln_{\theta }p_{x}{\;\ln}_{\theta }p_{z}+\ln_{\theta }p_{y}{\;\ln}_{\theta }p_{z}\right]\\ & \;\;\;\;\;+{\left(-\theta \right)}^{2}\left[\ln_{\theta }p_{x}{\;\ln}_{\theta }p_{y}{\;\ln}_{\theta }p_{z}\right], \end{array}$$

where square brackets enclose linear combinations of such products. Factors ${\left(-\theta \right)}^{k-1}$ in Equation (15) measure the strength of a many-body interaction among k=1, 2,…,K classes.

### 4.2. The Model

The replacement of $s_{BE}\left(\nu_{k}\right)$ in Equation (10) by $s_{\theta }\left(\nu_{k}\right)=\left(1+\nu_{k}\right)\ln_{\theta }\left(1+\nu_{k}\right)-\nu_{k}\ln_{\theta }\nu_{k\;}$ leads to a simple interaction-sensitive model. We have:

(16) $$\begin{array}{ll} \frac{\partial }{\partial \nu_{k}}[s_{\theta }\left(\nu_{k}\right)- & \nu_{k}\left(\beta \varphi_{BE}\left(\omega_{k},\lambda \right)+\alpha \right)]\\ & =\left(1-\theta \right)\left[\ln_{\theta }\left(1+\nu_{k}\right)-\ln_{\theta }\nu_{k}\right]-\left[\beta \varphi_{BE}\left(\omega_{k},\lambda \right)+\alpha \right]\\ & =\left[\frac{1-\theta }{\theta }\right]\left[\nu_{k}{}^{-\theta }-{\left(1+\nu_{k}\right)}^{-\theta }\right]-\left[\beta \varphi_{BE}\left(\omega_{k},\lambda \right)+\alpha \right]=0. \end{array}$$

**Figure 2:**
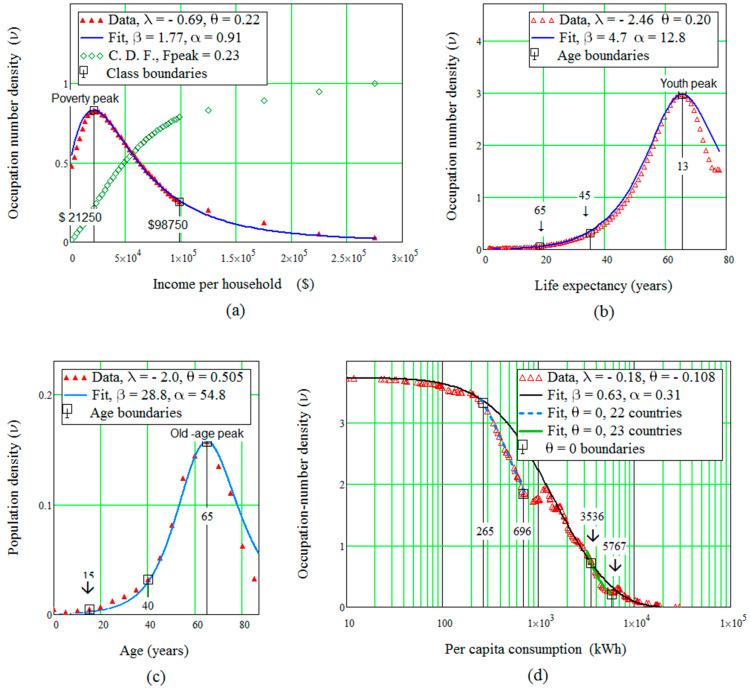
Data and fits for: (**a**) Income distribution. (**b**) Life expectancy. (**c**) Cancer incidence. (**d**) Electricity consumption.

Since resources are not expected to interact, Equations (6) and (14) give again the measure of equality and the value of λ, respectively. Plots of $\partial s_{\theta }\left(\nu_{k}\right)/\partial \nu_{k}$ as functions of $\varphi_{BE}\left(\omega ,\lambda \right)$ are close to a single straight line. We therefore obtain a first approximation to the numerical value of θ from the condition that it maximises the ordinate at the peak. An even better guess stems from a maximisation of Pearson’s correlation coefficient for the whole line; β and α result from subsequent linear regression on Equation (16) *once* λ and θ have been determined. Uncertainties due to multidimensional fits are thus avoided. Four such fits appear in Figure 2. They validate the asymmetric-entropic-quantum model, in particular conjectures C1, C1’ and C2.

Class boundaries and periods in human life are obtained from plots like those in Figure 1. Poverty, for instance, is objectively defined by the region $\left[0,\;\omega_{p}\right]$ under the data curve in Figure 2a.

### 4.3. Resource-Dependent Interactions

Annual *per capita* electricity consumption in Figure 2d is a special case; not only it is an example of rather unusual long-range interactions, it also requires two values of the θ-parameter, θ=−0.18 for an overall fit and θ=0, apparently noninteracting behaviour for two groups, of 22 and 23 countries. A possible explanation is that interactions between countries result mainly from their exchange of electricity, often carried out to optimise each country’s production systems. The poorest nations rely heavily on—and therefore interact strongly with—foreign production, which introduces correlations between countries. They would disappear for self-sufficient countries, which would thus form the first θ=0 group. Increasing production would make trade and therefore correlations to reappear, but they vanish again when import and export compensate each other, that is, the second group. The interplay of production, consumption and exchange imposes resource-dependent interactions and thereby several values of the θ-parameter.

Figure 2d illustrates another possible drawback: no data were available for the poorest countries (about 20 in number). As a result, the poverty peak is missing, unfortunately a rather common situation for an insufficient number of datapoints. The peak is assumed to coincide with the lowest electricity consumption in Figure 2d, and this is enough to furnish a rather acceptable fit for the data.

## 5. Conclusions

A simple probabilistic model fits data from two types of statistical phenomena, demographic and economical, through four different examples. Dynamical effects and interactions [13] are taken into account through their averages over definite periods of time, which allows good fits of real data. The model’s extension to other cases where inequality, symmetry and/or quantum behaviour are at work may be expected. It depends on the statistics of state occupation (of two types, F–D or B–E, the latter being the case of all examples in this paper), the resulting symmetry (two possibilities, though symmetric distributions are rather doubtful), the type of interactions (two levels in this paper, intraclass and interclass), their intensity (possibly variable, as for electric energy consumption) and their long or short range.

Results on welfare and universality support the proposal of a symmetry phase transition at Gi=1/3 between conceivable but unlikely symmetric distributions and realistic asymmetric laws. Phase transitions are thus possible not only in material systems, but also in societies, physiology and perhaps other correlated structures. Equation (16) provides expressions for their distribution laws, whereby specific regions under the distribution curve objectively define youth and oldness or poverty and wealth. Different forms of inequality are thus found to admit similar descriptions.

We obtain good quality fits to data by applying the paraphernalia of well-known, century-old statistical mechanics (states, entropy, Lagrange multipliers …) to social matters. The manner in which this is done is however atypical, particularly because symmetry is a relevant parameter, two entropies at least are at work, and nonquantum free-will individuals obey quantum statistics. Societies are considered as nonequilibrium, interacting, entropy-producing statistical systems. As a result: (1) individuals interact in at least two ways—intraclass and interclass; (2) inequality is an example of a “qualitative” quantity, for which generalisations of the present approach may be expected; (3) households and resources are clearly not quantal, but socially indistinguishable, wherefrom quantum statistics finally follows—quantum behaviour is not intrinsic, but results from the nature of occupied states; and (4) one of the entropies is the outcome of activities in an evolving society, the other simply measures inequality in the distribution of available resources, and furnishes a constraint on the former. In the general case, multiple entropies would be required to account for different types of inequality, and as many constraints on social entropy production S(ν) would result. Conjectures similar to C1’ and C2 would be required. The concepts of extropy, class interaction, multiple entropies and social free energy appear as efficient approaches to nonequilibrium evolving systems. Furthermore, diversity occurs in so many domains that similar methods may be expected to apply to energy production, environmental and other complex systems.

Only two supplementary parameters, θ due to the strength and range of interactions and λ related to inequality, suffice to transform the ideal-gas description of independent individuals into a predictive model of society. They result from the coordinates of a single point on the empirical distribution law, the peak. The additional information thus obtained may look rather scanty at first sight, were it not for a remark by E.T. Jaynes [30]: “Entropy as a concept may be regarded as a measure of our degree of ignorance as to the state of a system”. Our successful maximisation of entropy production implies then the safest possible assumption, that is, minimum social knowledge of economic and demographic statistical facts.
